# Use of Emerging Tobacco Products in the United States

**DOI:** 10.1155/2012/989474

**Published:** 2012-05-10

**Authors:** Robert McMillen, Jeomi Maduka, Jonathan Winickoff

**Affiliations:** ^1^Department of Psychology and Social Science Research Center Research Boulevard, Suite 103, Starkville, MS 39759, USA; ^2^Julius B. Richmond Center of Excellence, American Academy of Pediatrics, 141 Northwest Point Boulevard, Elk Grove Village, IL 60007, USA; ^3^University of Texas Southwestern Medical School, 5323 Harry Hines Boulevard, Dallas, TX 75390, USA; ^4^Massachusetts General Hospital, GH Center for Child and Adolescent Health Policy, 50 Staniford Street, Suite 901, Boston, MA 02114, USA

## Abstract

This paper provides the first nationally representative estimates for use of four emerging products. Addressing the issue of land-line substitution with cell phones, we used a mixed-mode survey to obtain two representative samples of US adults. Of 3,240 eligible respondents contacted, 74% completed surveys. In the weighted analysis, 13.6% have tried at least one emerging tobacco product; 5.1% snus; 8.8% waterpipe; 0.6% dissolvable tobacco products; 1.8% electronic nicotine delivery systems (ENDS) products. Daily smokers (25.1%) and nondaily smokers (34.9%) were the most likely to have tried at least one of these products, compared to former smokers (17.2%) and never smokers (7.7%), P<.001. 18.2% of young adults 18–24 and 12.8% of those >24 have tried one of these products, P<.01. In multivariable analysis, current daily (5.5, 4.3–7.6), nondaily (6.1, 4.0–9.3), and former smoking status (2.7, 2.1–3.6) remained significant, as did young adults (2.2, 1.6–3.0); males (3.5, 2.8–4.5); higher educational attainment; some college (2.7, 1.7–4.2); college degree (2.0, 1.3–3.3). Use of these products raises concerns about nonsmokers being at risk for nicotine dependence and current smokers maintaining their dependence. Greater awareness of emerging tobacco product prevalence and the high risk demographic user groups might inform efforts to determine appropriate public health policy and regulatory action.

## 1. Introduction

Recently, snus, dissolvable tobacco products, and electronic nicotine delivery systems (sometimes called “e-cigarettes” or ENDS) have been introduced to the US market, while waterpipes (hookah), especially in group social settings, have gained popularity [1]. Snus, dissolvables, ENDS, and waterpipes are often promoted as safer alternatives to traditional cigarettes and a potential way to decrease the harm caused by tobacco [2–4]. However, people who may never have smoked a cigarette or who had been addicted to nicotine in the past may be enticed to use tobacco by these alternative products, posing an individual and public health risk. Once in a tobacco using culture and exposed to nicotine, individuals may be at higher risk of regular cigarette use [5]. There is also the potential that current smokers may use these products as an alternative to cessation [6]. Polytobacco use among current smokers may increase levels of nicotine exposure and risk of persistent tobacco dependence relative to the exclusive use of cigarettes [7]. Despite these concerns, little is known about the use of these products among US adults. Although substantial research has examined other alternative tobacco products [8, 9], this is the first nationally representative study to examine the prevalence rates for these new emerging products. Data on the use of these emerging products is urgently needed as the FDA considers regulation of these products.

Snus is a smokeless tobacco product that does not require the user to spit. The tobacco in some snus has low concentrations of nitrosamines [2] and is marketed to smokers as a reduced harm product. Snus is also marketed in airports as a tobacco product that can be used in places where smoking is not allowed. If snus was to replace cigarette smoking entirely for an individual, it would be less harmful than cigarettes [3], but its most significant health risks may be in maintaining dependence to cigarettes and as a starter product for other forms of tobacco [10]. Proponents of the promotion of snus as a harm reduction policy look to the Swedish experience where studies have found that while snus use is increasing, smoking prevalence is declining [7]. However, promoting snus in the United States for harm reduction may reduce smoking cessation [11], perhaps because the USA already has ongoing tobacco control programs. Additionally, US tobacco companies market dual usage of both snus and cigarettes with slogans like: “When you cannot smoke, snus” [12].

Dissolvable tobacco products are also smokeless spit-less tobacco products. These products are typically flavored forms of finely milled tobacco and dissolve in the mouth. Like snus, these products are frequently marketed as forms of tobacco that can be used in places where smoking is prohibited or that are tobacco-free. To illustrate, one producer claims, “dissolvable tobacco has no boundaries, there are no locations or situations where you cannot use it, and nobody can tell you're using it” [13]. These products may also appeal to adolescents, due to the attractive packaging, flavoring, and dissolvable delivery system.

 ENDS are a category of products that deliver a vapor of nicotine and flavoring on inhalation [14]. These products are very new and are marketed as both cessation devices and an alternative to cessation [6]. ENDS come in a variety of tobacco, fruit, and food flavors, and, although they do not actually burn tobacco, some ENDS contain a light-emitting diode at the tip that resembles the burning end of a cigarette [6]. Because of their recent emergence, little research exists on their attractiveness.

Developed in India during the 1700s [15], a waterpipe is an instrument for inhaling charcoal tobacco smoke that has been cooled by passing through water. Although users may think that smoke inhaled from a waterpipe is safer than smoke from a cigarette, studies show that waterpipe use produces concentrations of carbon monoxide, nicotine, tar, and heavy metals at levels similar to, or higher than, cigarettes [1]. There is also the risk of infectious disease transmission, including herpes, from waterpipe mouthpieces [1]. Due to misperceptions that waterpipes are safe, and the use of these waterpipes in social settings, there is also the risk that nonsmokers might be attracted to waterpipe smoking. Most waterpipe users are intermittent cigarette smokers [16], which facilitates an opportunity in a tobacco-friendly environment for nonsmokers to become initiated to the cigarette smoking social culture as well [17].

The purpose of this study is to assess the prevalence of use of snus, waterpipe, dissolvable tobacco products, and ENDS. The prevalence of lifetime use and current use of these products by cigarette smoking status are examined, as well as other correlates of lifetime use. Results from this study can inform regulatory decisions about these products, while the identification of potential high risk demographic groups can guide clinical counseling efforts regarding the risks of any tobacco use. Finally, the use of these products among former smokers is examined to determine whether former smokers used these products as an acute form of nicotine replacement therapy to aid in cessation or used these products years after successfully quitting cigarettes.

## 2. Methods

### 2.1. Respondents

The Social Climate Survey of Tobacco Control (SCS-TC) is a nationally representative annual cross-sectional survey that contains items pertaining to normative beliefs, practices/policies, and knowledge regarding tobacco control. Previously, this survey has utilized a random-digit-dialing (RDD) frame of households with landline telephones [18, 19]. However, substitution of cell phones for landlines continues to increase and 27.8% of US households are currently wireless only [20]. Moreover, wireless substitution is particularly problematic for surveys of tobacco use, as smoking status, as well as age, region, and several other demographic factors vary by telephone status [20]. In order to reduce noncoverage issues arising from wireless substitution, mixed-mode, mixed-frame surveys representing national probability samples of adults were administered in 2010. The design included an RDD (mode 1) frame and an internet panel (mode 2) frame developed from a probability sample. The Institutional Review Board at Mississippi State University approved this study on July 30, 2010.

The mode 1 frame included households with listed and unlisted landline telephones. Telephone interviews with respondents were conducted in October and November 2010. Household telephone numbers were selected using RDD sampling procedures. Once a household was contacted, the adult to be interviewed was selected by asking to speak with the person in the household who is 18 years of age or older and who will have the next birthday. Five attempts were made to contact those selected adults who were not home.

The mode 2 frame included an online survey, administered in September and October of 2010 to a randomly selected sample from a nationally representative pre-established 50,000 member research panel [21, 22].

 The 50,000 panel members were randomly recruited by probability-based sampling, and households were provided with access to the Internet and hardware if needed in order to develop a panel that is representative of the entire US population [21]. This panel is based on a sampling frame which includes both listed and unlisted numbers, those without a landline telephone, and does not accept self-selected volunteers [21, 22]. Probability-based recruitment for the panel includes two frames. The RDD frame uses list-assisted RDD sampling techniques and the Address-Based Sampling (ABS) frame from the US Postal Service's Delivery Sequence File, which includes all households serviced by the US Postal Service [21]. The use of RDD and ABS frames for recruiting panel members provides sample coverage for 99% of US households [23]. A recent study examining this probability panel revealed that the panel's primary demographics are representative of the US Census [24]. Moreover, more than a hundred peer-reviewed papers have applied this survey methodology [25], including articles published in health journals [26–29]. 

Overall weights were computed in two steps. First, the two modes were weighted based upon 2009 US Census estimates to be representative of the US population. Second, three adjustments to these initial weights were computed to account for the overlap in the two samples. Weights from the mode 1 frame were multiplied by  .818 to adjust for the overlap (81.8% of households in the mode 2 frame had a landline). Composite adjustments were then computed to combine the two sampling frames. According to AAPOR [30], observations from two sampling frames with overlap may be combined using composite weights. Two compositing factors that sum to one are typically selected. Given that the effective sample sizes of the mode 1 frame and mode 2 frame are similar, the two compositing factors were set to 0.5. The weights of respondents who were represented in both sampling frames (i.e., landline owners) were multiplied by the compositing factor. In the final adjustment, a restandardized weight was computed so that the weighted sample size matched the sum for effective sample size for both independent frames.

### 2.2. Measures

Results are from data on a subset of the measures included in the SCS-TC. To assess current cigarette smoking status of respondents, respondents were asked, “Have you smoked at least 100 cigarettes in your entire life?”. Respondents who reported that they had were then asked, “Do you now smoke cigarettes every day, some days, or not at all?”. Respondents who reported that they have smoked at least 100 cigarettes and now smoke every day or some days were categorized as daily and nondaily smokers; respondents who had not smoked at least 100 cigarettes were categorized as never smokers; and respondents who reported that they have smoked at least 100 cigarettes, but no longer smokers were categorized as former smokers.

One set of items assessed lifetime use of emerging tobacco products. Which of the following products have you tried, even just one time? (1) Smokeless tobacco, (2) snus, such as Camel or Marlboro snus, (3) roll-your-own cigarettes, (4) smoking tobacco from a hookah or a waterpipe, (5) dissolvable tobacco products like Ariva/Stonewall/Camel/Camel Orbs/Camel sticks, (6) electronic cigarettes or E-cigarettes, such as Ruyan or NJOY. Respondents who had tried a product were asked if they had used that product in the past 30 days. Those who had were considered to be current users (analyses in this paper were limited to products that are new to the US market or that have recently gained popularity). 

Sociodemographic variables included four categories for region (determined by the US Census regions), three categories for self-reported race (white, single race; black, single race; and all other responses), two categories for age (18–24 and 25+), and sex. The two age categories were selected in order to determine if younger adults were the most susceptible to using these emerging products.

### 2.3. Analyses

Chi-square tests were used to examine smoking status and sociodemographic characteristics among lifetime and current users of these nicotine-containing products. For the analyses by smoking status, post hoc multiple comparisons of never smokers versus former smokers and nondaily smokers versus daily smokers were conducted with an adjusted alpha level set at  0.05/6 or  0.008.

Multivariable analysis was applied to assess the relationship of smoking status, age, and other sociodemographic characteristics with lifetime use. To explore the possibility that adults were using these products as a form of nicotine replacement therapy, chi-square analyses were used to compare use of at least one of these products among former smokers by the length of time since cessation.

In order to address the possibility that former smokers used one of these emerging products prior to cessation, chi-square tests were used to examine use of these products among former smokers who quit less than a year ago, one to five years ago, five to 10 years, and more than 10 years. Although our data do not allow us to directly determine whether use of these emerging products occurred before or after smoking cessation, these analyses will provide insight into whether smoking cessation or use of an emerging product occurred first. It is doubtful that someone who quit smoking more than five years ago used one of these emerging products prior to cessation.

## 3. Results

In mode 1, of 2,128 eligible respondents contacted, 1,504 (70.7%) completed surveys [30]. For the mode 2 frame, 2,272 panelists were randomly drawn from the probability panel [31]; 1,736 responded to the invitation, yielding a final stage completion rate [26] of 67.5% percent. Length of time on the panel for the mode 2 frame ranged from  0.09 to 11.08 years, with a median length of time on the panel of 2.29 years.  Table 1 shows the demographic characteristics of the overall sample. 

### 3.1. Lifetime Users of Emerging Tobacco Products

Although most adults have not tried any of these tobacco products (86.4%), some adults have tried a waterpipe (8.8%) or snus (5.1%). Fewer adults have tried an ENDS product (1.8%) or dissolvable tobacco products (0.6%). Nondaily (34.9%) and daily smokers (25.1%) were the most likely to have tried each of these tobacco products (*P* < .001); however, some nonsmokers had tried at least one of these products (see Table 2). Among the nonsmokers, former smokers (17.2%) were more likely than never smokers (7.7%) to have used at least one of these tobacco products (*P* < .001). Use of these products also varied across nondaily and daily smokers. Although daily smokers (12.9%) were more likely to have tried snus than nondaily smokers (4.1%), *P* = .003, ever use of waterpipe was higher among nondaily smokers 26.0% than daily smokers (12.9%), *P* < .001.

Age, sex, region, race, and education were also significantly associated with lifetime use for at least one of these products (see Table 2). Younger adults were more likely than older adults to have tried snus and water pipe (8.0% versus 4.6%, 12.3% versus 8.2%, resp., *P* < .01); males were more likely than females to have tried each of these products (see Table 2), with the exception of electronic cigarettes.

Table 3 presents the odds ratios from a logistic regression of lifetime use of at least one of these emerging products on smoking status, region, race, age, education, and sex (the pattern of results did not change when this logistic regression model was replicated with sample frame included as a predictor). Most notable was the strong association between use of emerging tobacco products with young age, male gender, and higher education when controlling for smoking status.

### 3.2. Current Users of Emerging Tobacco Products

Current use of these tobacco products was rare (current use did not exceed 1% for any of these products). However, current use among adults who had ever used these products was nontrivial, snus (14.4%), waterpipe (11.4%), and ENDS (19.7%). Conversely, current use of dissolvable tobacco products among ever users was less than one percent.

### 3.3. Cessation and Use of Emerging Tobacco Products

Of significant concern is the use of these products by former smokers after they had successfully quit smoking cigarettes. However, it is possible that some former smokers used these emerging tobacco products as a form of nicotine replacement therapy to help them quit, or simply tried one of these products before they quit smoking cigarettes. To address this possibility, we compared the use of these products among former smokers who quit smoking less than 1 year ago (7.2%), one to five years ago (17.1%), five to 10 years (14.6%), and more than 10 years (61.0%). People who had quit smoking more recently (<1 year ago) were the most likely to report having tried one of these products 32.1%; 27.1%; 14.9%; 13.5%, respectively (*P* < .001 for trend). However, the distant former smokers, defined as >5 years quit, accounted for 59.7% of those who had every tried one of these products.

## 4. Discussion

There are many concerns regarding emerging tobacco products; this is the first study to examine use of these products in a nationally representative sample. Our findings demonstrate that more than one in 10 US adults have tried at least one emerging tobacco product. Although overall current use of these products was low, a nontrivial percentage of people who had tried snus, waterpipe, or ENDS were current users. More people have tried a waterpipe than snus or ENDS, however ENDS and snus are newer to the US market. Daily and nondaily smokers were the most likely to have tried each of these products. Furthermore, nondaily smokers are the most likely to have tried a waterpipe. 

Our study also demonstrates that lifetime use of these products is more common among males than females and younger adults than older adults, whereas lifetime use is lowest among adults living in the southern region of the US Contrary to cigarette use patterns, higher levels of education are associated with higher use of at least one of these emerging products. This relationship is the inverse of the trend toward decreased cigarette use in the higher educated demographic groups, suggesting that emerging products may have the capacity to “re-normalize” tobacco use in a demographic that has had significant denormalization of tobacco use previously.

All forms of tobacco are potentially harmful but the use of these emerging products is concerning for at least four additional reasons. First, the use of these products by people who have never smoked cigarettes may lead to desensitization to the concept of using tobacco products in general. Tolerance to tobacco and less normative resistance to tobacco could lead to future use of cigarettes. In addition, these products contain nicotine and will therefore start the upregulation of nicotine receptors in the reward centers of the brain, setting up the potential for nicotine addiction and a facilitated leap to the cigarette [5]. Second, people who have quit smoking may relapse to nicotine addiction after using these products. Recent former smokers are particularly susceptible to relapse early on, whereas distant former smokers may still relapse back to smoking cigarettes especially when using other tobacco products [32]. Third, current smokers may use these products as an alternative to cessation [33]. Although replacing cigarettes with these other tobacco delivery devices might be beneficial, the risk of relapse to cigarette smoking may be elevated compared to people who overcome their addition without continuing the behavioral act of cigarette use itself. And fourth, the lifetime prevalence of using waterpipe among nondaily smokers is more than 25% and substantially higher than among daily smokers and nonsmokers. Polytobacco use among these nondaily smokers may also increase levels of nicotine exposure and risk of persistent tobacco dependence relative to the exclusive use of cigarettes [7].

The higher lifetime prevalence rate for use of these products among young adults, males, more educated adults, and residents outside of the southern region suggest that public health strategies should prioritize preventing additional or further use of these products in these populations, while maintaining lower lifetime prevalence rates in other groups. Almost 20% of young adults have tried at least one of these emerging tobacco products.

There are at least two unique strengths of this study. These are the first nationally representative data on the prevalence of use of these emerging products. This information can help to inform efforts to determine the need for regulatory protections. Furthermore, these findings are based on a nationally-representative sample of US adults obtained from a mixed-mode frame that substantially reduces concerns of the increasing bias in RDD surveys arising from noncoverage due to wireless substitution. However, this study is subject to at least five limitations.

First, although the mixed-mode design substantially reduced noncoverage bias compared to an RDD design by including respondents who did not have a landline telephone in their home, it is possible that the dual sampling frame did not entirely eliminate noncoverage issues. The use of the internet panel raises some concern about the representativeness of the sample. However, several comparison studies have demonstrated that this approach yields results comparable to well-designed RDD surveys, in terms of demographics and outcome variables [24, 34]. Chang and Krosnick compared findings from this internet panel, an RDD survey, and a nonprobability internet survey (Harris Interactive Internet Panel). The RDD and internet panel probability samples were found to be more representative than the nonprobability internet sample. Compared to the RDD sample, this internet probability panel demonstrated less evidence of survey satisficing and social desirability than the RDD survey, frequent concerns with tobacco use survey research [34]. More recently, Yeager and colleagues conducted a similar comparison study that also included benchmarks from the National Health Interview Survey and the Current Population Survey [24]. Again, this internet panel probability sample was comparable to these large government surveys in terms of demographic, behavioral, and attitudinal benchmarks and were found to be more representative than seven different nonprobability internet surveys [24].

Second, ongoing engagement might lead to panel conditioning, and thereby reduce data reliability if respondents develop a “time-in-sample bias” due to increased experience with completing surveys. However, results from the primary analyses did not change with the inclusion of a variable that measured time on the panel. (For the mode 2 frame, analyses presented in Tables 2 and 3 were replicated with the inclusion of a variable that measured length of time on the panel. The pattern of results did not change, and no evidence of a “time-on-panel bias” was detected.)

Third, the cumulative response rate for the mode 2 frame is significantly lower than the response rate from mode 1. However, it is important to note the differences between an RDD telephone sample and a probability-based internet panel. An online panel is composed of people recruited at different times and, more importantly, committed to answer many surveys for a period of time and not just that single survey. Further, panelists must also complete profiling surveys in order to become members of the panel. These differences are reflected in the recruitment and profile rates reported above. These differences make directly comparing response rates between one-time surveys and panel surveys difficult and perhaps not illuminating.

When considering the first three limitations, it is worth comparing estimates from the 2010 SCS-TC to those from a large-scale national survey. Both the SCS-TC and the National Health Interview Survey (NHIS) [35] assess current smoking status using the same survey items, and produced very similar estimates (SCS-TC, 18.3%, NHIS, 19.4%). Thus this prevalence estimate from the SCS-TC is comparable to that from one of the principal sources of information about the health of the US population.

The fourth limitation relates to whether any of the recent former smokers had quit cigarettes because of these emerging tobacco products, or, rather, had used these products after successfully quitting. Obviously those former smokers who quit before these products emerged in the US market did not use these products as a cessation strategy, but this is an area for future study among people who have recently quit smoking.

 The fifth limitation concerns the cross-sectional nature and scope of these data. As noted above, it is not possible from this survey to determine when adults, particularly former smokers, tried these products. Moreover, an expanded pool of survey items that assessed when and under what scenarios people used these products would provide more conclusive insight into the risks that these products pose. Further studies should include more detailed items to examine perceptions and use of these emerging products among adolescents and young adults who are closer to the median age of cigarette smoking initiation.

An expanding pool of tobacco products with little or no regulation may increase the overall number of individuals who become nicotine dependent and later use cigarettes. This study demonstrates that some young adults, distant former, and never cigarette smokers have used these emerging nicotine-containing tobacco products, suggesting a need to restrict how and to whom these products are marketed, sold, and used. Furthermore, clinicians need to be aware of emerging tobacco products, both to better screen high risk demographic groups, and to offer counseling about the risks of these products as another form of tobacco use.

## Figures and Tables

**Table 1:** Demographic characteristics of respondents (unweighted *N* = 3,240).

Demographic variable	Overall *N *	Overall weighted percent	Mode 1 frame unweighted percent	Mode 2 frame unweighted percent
Smoking status				
Never smoker	1,802	56.9%	56.9%	52.3%
Former smoker	787	24.8%	28.3%	28.3%
Nondaily smoker	146	4.6%	1.6%	4.0%
Daily smoker	434	13.7%	13.2%	15.4%
Region				
Northeast	404	12.6%	18.7%	18.9%
Midwest	589	18.4%	25.5%	22.4%
South	1,203	37.6%	39.5%	37.0%
West	1,007	31.4%	16.4%	21.7%
Race				
White	2,346	74.2%	87.2%	73.8%
African American	364	11.5%	10.0%	8.5%
Other	454	14.3%	2.7%	17.7%
Age				
18–24	440	13.7%	8.3%	8.1%
25+	2,763	86.3%	91.7%	91.9%
Education				
Not a high school graduate	291	9.2%	5.6%	11.2%
High school graduate	903	28.5%	28.6%	29.0%
Some college	929	29.3%	25.9%	28.0%
College graduate	1,044	33.0%	40.0%	31.7%
Sex				
Female	1,523	52.3%	36.2%	46.7%
Male	1,675	47.6%	63.8%	53.3%

**Table 2:** Ever use of nicotine products by respondent characteristics.

	Snus	Waterpipe	Dissolvable tobacco products	ENDS	At least one of these products
Overall	5.1% (*n* = 162)	8.8% (*n* = 281)	0.6% (*n* = 20)	1.8% (*n* = 56)	13.6% (*n* = 435)

Smoking status	*P* < .001	*P* < .001	*P* = .001	*P* < .001	*P* < .001
Never smokers	2.7% (*n* = 48)	5.4% (*n* = 97)	0.2% (*n* = 3)	0.3% (*n* = 6)	7.7% (*n* = 139)
Former smokers	6.5% (*n* = 51)	11.4% (*n* = 90)	1.1% (*n* = 9)	1.5% (*n* = 12)	17.2% (*n* = 135)
Nondaily smokers	4.1% (*n* = 6)	26.0% (*n* = 38)	2.7% (*n* = 4)	8.2% (*n* = 12)	34.9% (*n* = 51)
Daily smokers	12.9% (*n* = 56)	12.9% (*n* = 56)	0.9% (*n* = 4)	6.2% (*n* = 27)	25.1% (*n* = 109)

Region	*P* = .076	*P* < .001	*P* = .520	*P* = .396	*P* < .001
Northeast	3.2% (*n* = 13)	12.6% (*n* = 51)	0.2% (*n* = 1)	2.7% (*n* = 11)	15.6% (*n* = 63)
Midwest	6.5% (*n* = 38)	10.0% (*n* = 59)	0.5% (*n* = 3)	1.4% (*n* = 8)	15.1% (*n* = 89)
South	4.5% (*n* = 54)	4.8% (*n* = 58)	0.6% (*n* = 7)	1.6% (*n* = 19)	9.5% (*n* = 114)
West	5.7% (*n* = 57)	11.2% (*n* = 113)	0.9% (*n* = 9)	1.9% (*n* = 19)	16.9% (*n* = 170)

Race	*P* = .372	*P* = .006	*P* = .786	*P* = .971	*P* = .002
White	5.3% (*n* = 124)	9.5% (*n* = 222)	0.6% (*n* = 15)	1.7% (*n* = 41)	14.6% (*n* = 343)
Black	3.6% (*n* = 13)	4.4% (*n* = 16)	0.8% (*n* = 3)	1.9% (*n* = 7)	7.7% (*n* = 28)
Other	4.8% (*n* = 22)	9.5% (*n* = 43)	0.4% (*n* = 2)	1.8% (*n* = 8)	13.2% (*n* = 60)

Age	*P* = .003	*P* = .005	*P* = .626	*P* = .195	*P* = .002
18–24	8.0% (*n* = 35)	12.3% (*n* = 54)	0.5% (*n* = 2)	2.5% (*n* = 11)	18.2% (*n* = 80)
25+	4.6% (*n* = 128)	8.2% (*n* = 227)	0.7% (*n* = 18)	1.6% (*n* = 45)	12.8% (*n* = 355)

Sex	*P* < .001	*P* < .001	*P* < .001	*P* = .087	*P* < .001
Males	8.5% (*n* = 130)	13.6% (*n* = 208)	1.2% (*n* = 18)	2.2% (*n* = 33)	20.8% (*n* = 317)
Females	2.0% (*n* = 33)	4.4% (*n* = 74)	0.1% (*n* = 2)	1.4% (*n* = 23)	7.0% (*n* = 118)

Education	*P* < .001	*P* < .001	*P* = .107	*P* < .001	*P* < .001
Less than HS	3.8% (*n* = 11)	8.2% (*n* = 24)	0.0% (*n* = 0)	0.7% (*n* = 2)	10.3% (*n* = 30)
High school	7.8% (*n* = 70)	4.9% (*n* = 44)	0.3% (*n* = 3)	1.7% (*n* = 15)	12.7% (*n* = 115)
Some college	4.8% (*n* = 45)	12.8% (*n* = 119)	1.1% (*n* = 10)	3.7% (*n* = 34)	18.2% (*n* = 169)
College degree	3.2% (*n* = 33)	8.9% (*n* = 93)	0.7% (*n* = 7)	0.5% (*n* = 5)	11.3% (*n* = 118)

**Table 3:** Final logistic regression model showing odds of having tried a waterpipe, snus, or ENDS (*N* = 3,158).

Predictors	Have tried one of these products adjusted OR (95% confidence interval)
Smoking status	
Former smoker	2.71 (2.06, 3.56)
Nondaily smoker	6.13 (4.02, 9.33)
Daily smoker	5.53 (4.03, 7.58)
Region	
Northeast	1.68 (1.16, 2.42)
Midwest	1.65 (1.20, 2.28)
West	1.80 (1.36, 2.39)
Age	
18–24	2.18 (1.60, 2.97)
Sex	
Males	3.51 (2.77, 4.45)
Education	
High school	1.58 (.99, 2.51)
Some college	2.67 (1.69, 4.22)
College degree	2.04 (1.26, 3.30)

Model also included race, not significant. Reference groups were as follows: never smokers, south region, 25 years of age and older, females, and no high school degree.
